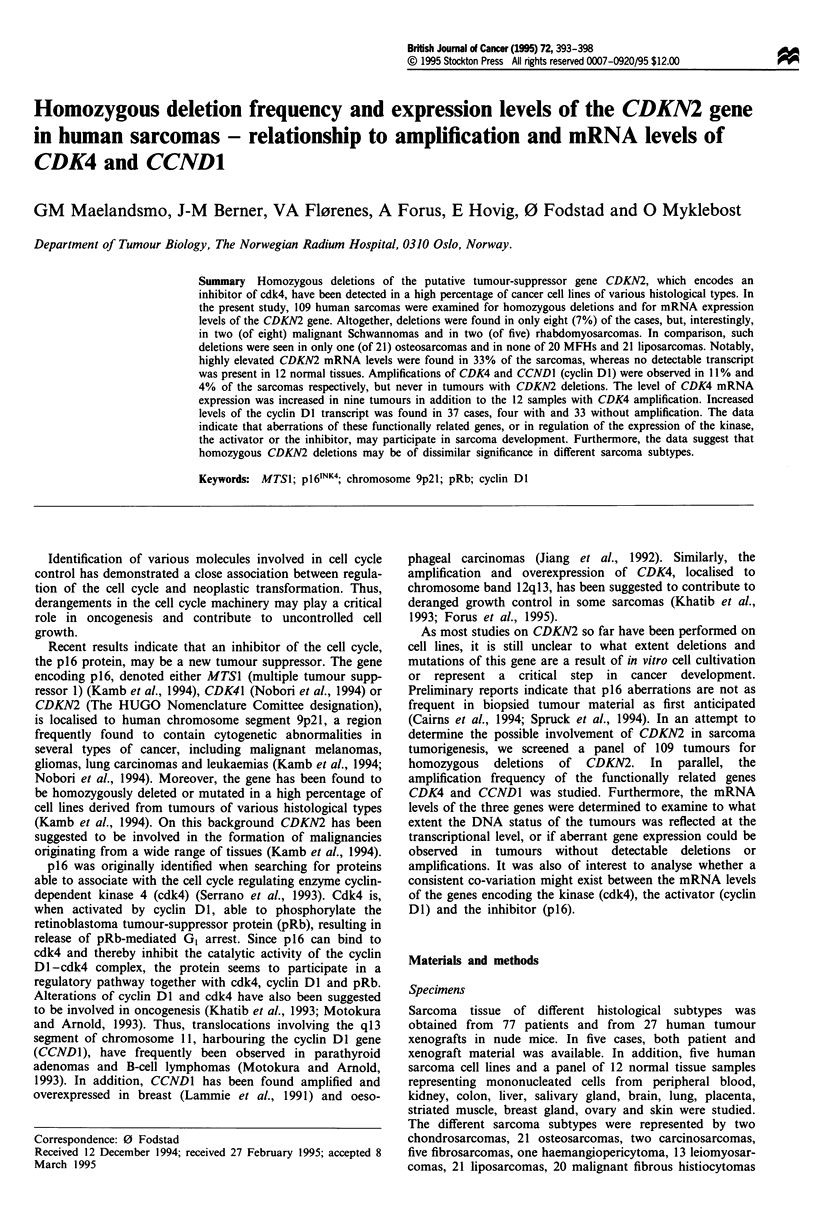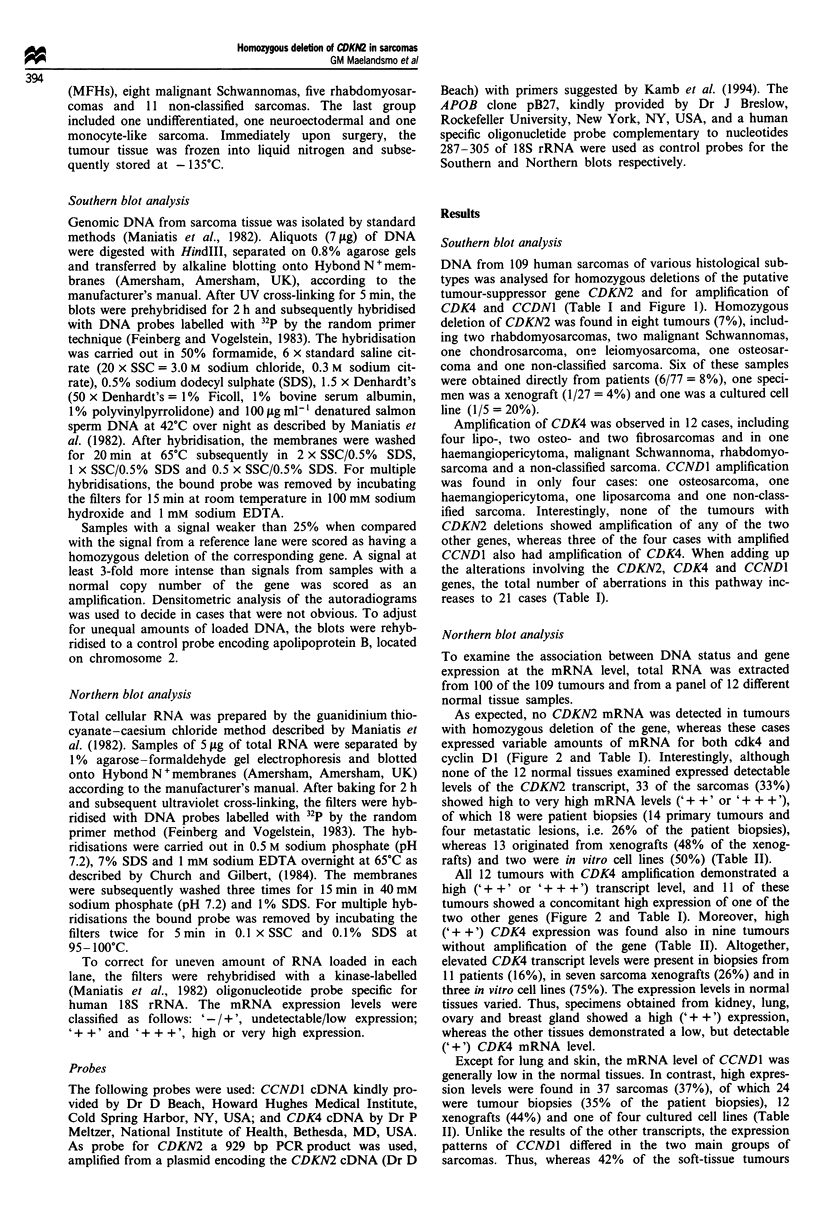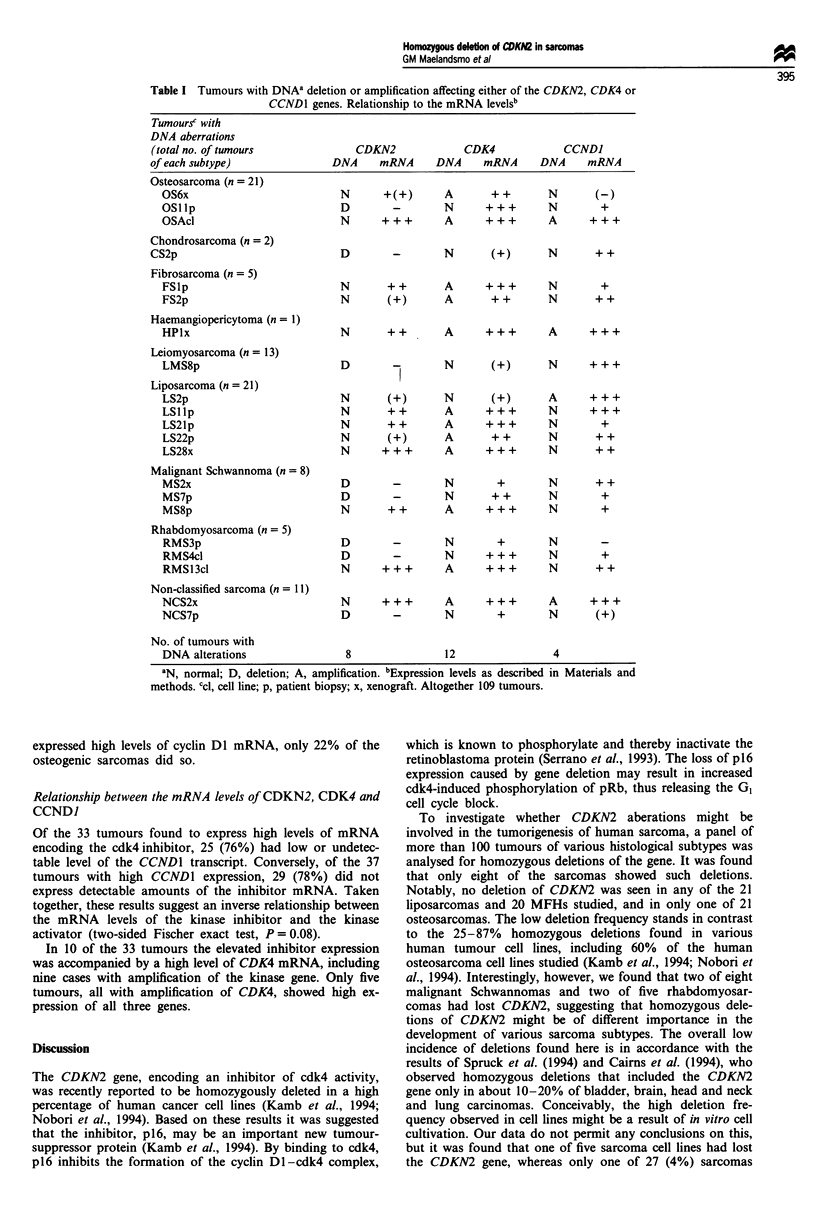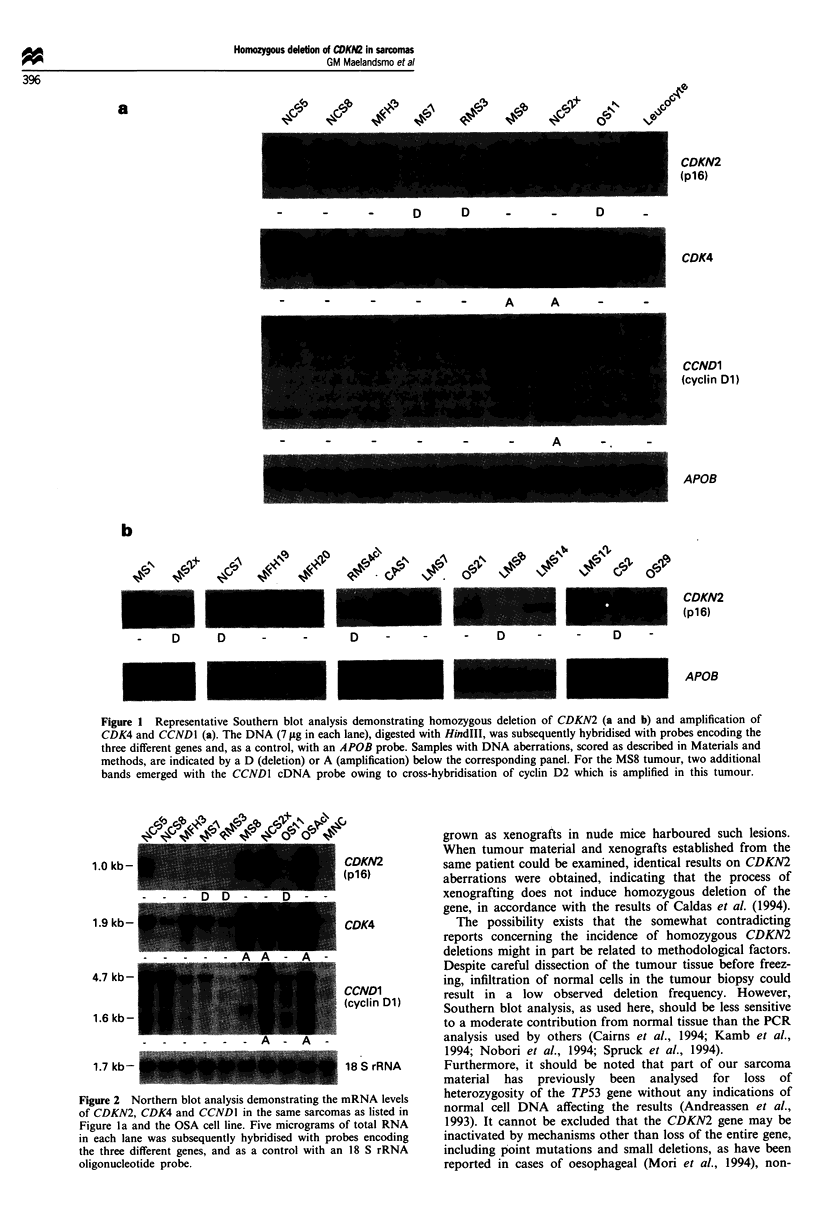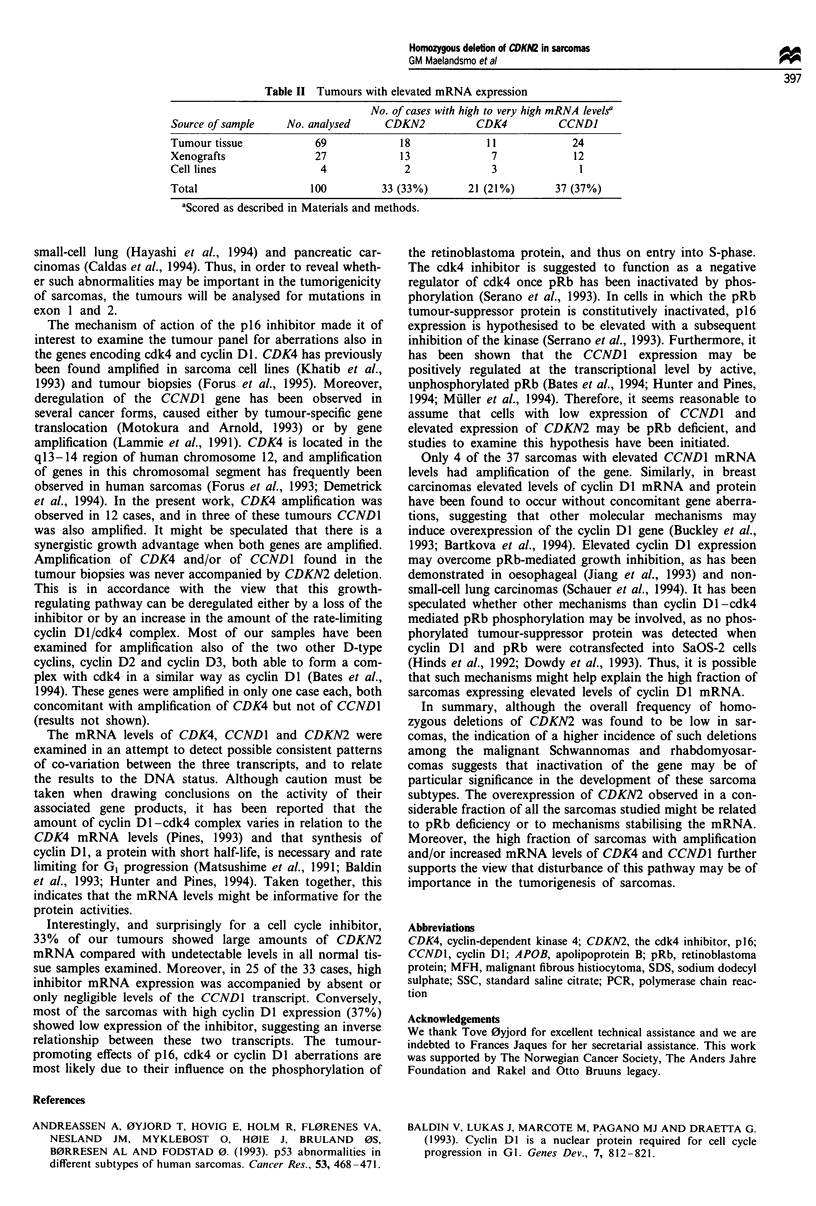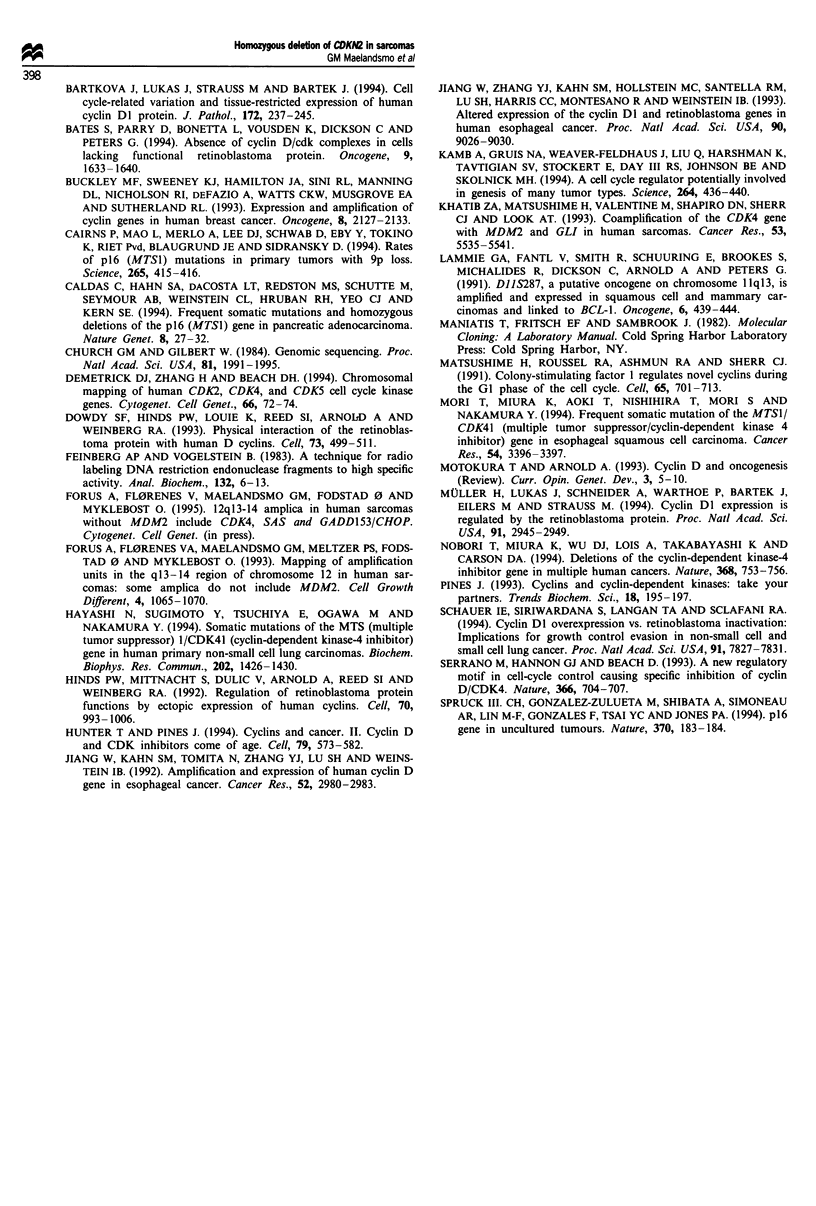# Homozygous deletion frequency and expression levels of the CDKN2 gene in human sarcomas--relationship to amplification and mRNA levels of CDK4 and CCND1.

**DOI:** 10.1038/bjc.1995.344

**Published:** 1995-08

**Authors:** G. M. Maelandsmo, J. M. Berner, V. A. Flørenes, A. Forus, E. Hovig, O. Fodstad, O. Myklebost

**Affiliations:** Department of Tumour Biology, Norwegian Radium Hospital, Oslo.

## Abstract

**Images:**


British Journal of Cancer (1995) 72, 393–398
© 1995 Stockton Press All rights reserved 0007-0920/95 $12.00

### Homozygous deletion frequency and expression levels of the *CDKN2* gene in human sarcomas – relationship to amplification and mRNA levels of *CDK4* and *CCND1*

GM Maelandsmo, J-M Berner, VA Flørenes, A Forus, E Hovig, Ø Fodstad and O Myklebost

*Department of Tumour Biology, The Norwegian Radium Hospital, 0310 Oslo, Norway.*

**Summary** Homozygous deletions of the putative tumour-suppressor gene *CDKN2*, which encodes an inhibitor of cdk4, have been detected in a high percentage of cancer cell lines of various histological types. In the present study, 109 human sarcomas were examined for homozygous deletions and for mRNA expression levels of the *CDKN2* gene. Altogether, deletions were found in only eight (7%) of the cases, but, interestingly, in two (of eight) malignant Schwannomas and in two (of five) rhabdomyosarcomas. In comparison, such deletions were seen in only one (of 21) osteosarcomas and in none of 20 MFHs and 21 liposarcomas. Notably, highly elevated *CDKN2* mRNA levels were found in 33% of the sarcomas, whereas no detectable transcript was present in 12 normal tissues. Amplifications of *CDK4* and *CCND1* (cyclin D1) were observed in 11% and 4% of the sarcomas respectively, but never in tumours with *CDKN2* deletions. The level of *CDK4* mRNA expression was increased in nine tumours in addition to the 12 samples with *CDK4* amplification. Increased levels of the cyclin D1 transcript was found in 37 cases, four with and 33 without amplification. The data indicate that aberrations of these functionally related genes, or in regulation of the expression of the kinase, the activator or the inhibitor, may participate in sarcoma development. Furthermore, the data suggest that homozygous *CDKN2* deletions may be of dissimilar significance in different sarcoma subtypes.

**Keywords:** *MTS1*; p16$^{INK4}$; chromosome 9p21; pRb; cyclin D1

Identification of various molecules involved in cell cycle control has demonstrated a close association between regulation of the cell cycle and neoplastic transformation. Thus, derangements in the cell cycle machinery may play a critical role in oncogenesis and contribute to uncontrolled cell growth.

Recent results indicate that an inhibitor of the cell cycle, the p16 protein, may be a new tumour suppressor. The gene encoding p16, denoted either *MTS1* (multiple tumour suppressor 1) (Kamb *et al.*, 1994), *CDK4I* (Nobori *et al.*, 1994) or *CDKN2* (The HUGO Nomenclature Comittee designation), is localised to human chromosome segment 9p21, a region frequently found to contain cytogenetic abnormalities in several types of cancer, including malignant melanomas, gliomas, lung carcinomas and leukaemias (Kamb *et al.*, 1994; Nobori *et al.*, 1994). Moreover, the gene has been found to be homozygously deleted or mutated in a high percentage of cell lines derived from tumours of various histological types (Kamb *et al.*, 1994). On this background *CDKN2* has been suggested to be involved in the formation of malignancies originating from a wide range of tissues (Kamb *et al.*, 1994).

p16 was originally identified when searching for proteins able to associate with the cell cycle regulating enzyme cyclin-dependent kinase 4 (cdk4) (Serrano *et al.*, 1993). Cdk4 is, when activated by cyclin D1, able to phosphorylate the retinoblastoma tumour-suppressor protein (pRb), resulting in release of pRb-mediated $G_1$ arrest. Since p16 can bind to cdk4 and thereby inhibit the catalytic activity of the cyclin D1–cdk4 complex, the protein seems to participate in a regulatory pathway together with cdk4, cyclin D1 and pRb. Alterations of cyclin D1 and cdk4 have also been suggested to be involved in oncogenesis (Khatib *et al.*, 1993; Motokura and Arnold, 1993). Thus, translocations involving the q13 segment of chromosome 11, harbouring the cyclin D1 gene (*CCND1*), have frequently been observed in parathyroid adenomas and B-cell lymphomas (Motokura and Arnold, 1993). In addition, *CCND1* has been found amplified and overexpressed in breast (Lammie *et al.*, 1991) and oesophageal carcinomas (Jiang *et al.*, 1992). Similarly, the amplification and overexpression of *CDK4*, localised to chromosome band 12q13, has been suggested to contribute to deranged growth control in some sarcomas (Khatib *et al.*, 1993; Forus *et al.*, 1995).

As most studies on *CDKN2* so far have been performed on cell lines, it is still unclear to what extent deletions and mutations of this gene are a result of *in vitro* cell cultivation or represent a critical step in cancer development. Preliminary reports indicate that p16 aberrations are not as frequent in biopsied tumour material as first anticipated (Cairns *et al.*, 1994; Spruck *et al.*, 1994). In an attempt to determine the possible involvement of *CDKN2* in sarcoma tumorigenesis, we screened a panel of 109 tumours for homozygous deletions of *CDKN2*. In parallel, the amplification frequency of the functionally related genes *CDK4* and *CCND1* was studied. Furthermore, the mRNA levels of the three genes were determined to examine to what extent the DNA status of the tumours was reflected at the transcriptional level, or if aberrant gene expression could be observed in tumours without detectable deletions or amplifications. It was also of interest to analyse whether a consistent co-variation might exist between the mRNA levels of the genes encoding the kinase (cdk4), the activator (cyclin D1) and the inhibitor (p16).

### Materials and methods

### *Specimens*

Sarcoma tissue of different histological subtypes was obtained from 77 patients and from 27 human tumour xenografts in nude mice. In five cases, both patient and xenograft material was available. In addition, five human sarcoma cell lines and a panel of 12 normal tissue samples representing mononucleated cells from peripheral blood, kidney, colon, liver, salivary gland, brain, lung, placenta, striated muscle, breast gland, ovary and skin were studied. The different sarcoma subtypes were represented by two chondrosarcomas, 21 osteosarcomas, two carcinosarcomas, five fibrosarcomas, one haemangiopericytoma, 13 leiomyosarcomas, 21 liposarcomas, 20 malignant fibrous histiocytomas

Correspondence: Ø Fodstad
Received 12 December 1994; received 27 February 1995; accepted 8 March 1995

(MFHs), eight malignant Schwannomas, five rhabdomyosarcomas and 11 non-classified sarcomas. The last group included one undifferentiated, one neuroectodermal and one monocyte-like sarcoma. Immediately upon surgery, the tumour tissue was frozen into liquid nitrogen and subsequently stored at −135°C.

*Southern blot analysis*

Genomic DNA from sarcoma tissue was isolated by standard methods (Maniatis *et al.*, 1982). Aliquots (7 μg) of DNA were digested with *Hin*dIII, separated on 0.8% agarose gels and transferred by alkaline blotting onto Hybond $N^+$ membranes (Amersham, Amersham, UK), according to the manufacturer's manual. After UV cross-linking for 5 min, the blots were prehybridised for 2 h and subsequently hybridised with DNA probes labelled with $^{32}P$ by the random primer technique (Feinberg and Vogelstein, 1983). The hybridisation was carried out in 50% formamide, 6 × standard saline citrate (20 × SSC = 3.0 M sodium chloride, 0.3 M sodium citrate), 0.5% sodium dodecyl sulphate (SDS), 1.5 × Denhardt's (50 × Denhardt's = 1% Ficoll, 1% bovine serum albumin, 1% polyvinylpyrrolidone) and 100 $\mu g\ ml^{-1}$ denatured salmon sperm DNA at 42°C over night as described by Maniatis *et al.* (1982). After hybridisation, the membranes were washed for 20 min at 65°C subsequently in 2 × SSC/0.5% SDS, 1 × SSC/0.5% SDS and 0.5 × SSC/0.5% SDS. For multiple hybridisations, the bound probe was removed by incubating the filters for 15 min at room temperature in 100 mM sodium hydroxide and 1 mM sodium EDTA.

Samples with a signal weaker than 25% when compared with the signal from a reference lane were scored as having a homozygous deletion of the corresponding gene. A signal at least 3-fold more intense than signals from samples with a normal copy number of the gene was scored as an amplification. Densitometric analysis of the autoradiograms was used to decide in cases that were not obvious. To adjust for unequal amounts of loaded DNA, the blots were rehybridised to a control probe encoding apolipoprotein B, located on chromosome 2.

*Northern blot analysis*

Total cellular RNA was prepared by the guanidinium thiocyanate–caesium chloride method described by Maniatis *et al.* (1982). Samples of 5 μg of total RNA were separated by 1% agarose–formaldehyde gel electrophoresis and blotted onto Hybond $N^+$ membranes (Amersham, Amersham, UK) according to the manufacturer's manual. After baking for 2 h and subsequent ultraviolet cross-linking, the filters were hybridised with DNA probes labelled with $^{32}P$ by the random primer method (Feinberg and Vogelstein, 1983). The hybridisations were carried out in 0.5 M sodium phosphate (pH 7.2), 7% SDS and 1 mM sodium EDTA overnight at 65°C as described by Church and Gilbert, (1984). The membranes were subsequently washed three times for 15 min in 40 mM sodium phosphate (pH 7.2) and 1% SDS. For multiple hybridisations the bound probe was removed by incubating the filters twice for 5 min in 0.1 × SSC and 0.1% SDS at 95–100°C.

To correct for uneven amount of RNA loaded in each lane, the filters were rehybridised with a kinase-labelled (Maniatis *et al.*, 1982) oligonucleotide probe specific for human 18S rRNA. The mRNA expression levels were classified as follows: '−/+', undetectable/low expression; '++' and '+++', high or very high expression.

*Probes*

The following probes were used: *CCND*1 cDNA kindly provided by Dr D Beach, Howard Hughes Medical Institute, Cold Spring Harbor, NY, USA; and *CDK*4 cDNA by Dr P Meltzer, National Institute of Health, Bethesda, MD, USA. As probe for *CDKN*2 a 929 bp PCR product was used, amplified from a plasmid encoding the *CDKN*2 cDNA (Dr D Beach) with primers suggested by Kamb *et al.* (1994). The *APOB* clone pB27, kindly provided by Dr J Breslow, Rockefeller University, New York, NY, USA, and a human specific oligonucletide probe complementary to nucleotides 287–305 of 18S rRNA were used as control probes for the Southern and Northern blots respectively.

### Results

*Southern blot analysis*

DNA from 109 human sarcomas of various histological subtypes was analysed for homozygous deletions of the putative tumour-suppressor gene *CDKN*2 and for amplification of *CDK*4 and *CCDN*1 (Table I and Figure 1). Homozygous deletion of *CDKN*2 was found in eight tumours (7%), including two rhabdomyosarcomas, two malignant Schwannomas, one chondrosarcoma, one leiomyosarcoma, one osteosarcoma and one non-classified sarcoma. Six of these samples were obtained directly from patients (6/77 = 8%), one specimen was a xenograft (1/27 = 4%) and one was a cultured cell line (1/5 = 20%).

Amplification of *CDK*4 was observed in 12 cases, including four lipo-, two osteo- and two fibrosarcomas and in one haemangiopericytoma, malignant Schwannoma, rhabdomyosarcoma and a non-classified sarcoma. *CCND*1 amplification was found in only four cases: one osteosarcoma, one haemangiopericytoma, one liposarcoma and one non-classified sarcoma. Interestingly, none of the tumours with *CDKN*2 deletions showed amplification of any of the two other genes, whereas three of the four cases with amplified *CCND*1 also had amplification of *CDK*4. When adding up the alterations involving the *CDKN*2, *CDK*4 and *CCND*1 genes, the total number of aberrations in this pathway increases to 21 cases (Table I).

*Northern blot analysis*

To examine the association between DNA status and gene expression at the mRNA level, total RNA was extracted from 100 of the 109 tumours and from a panel of 12 different normal tissue samples.

As expected, no *CDKN*2 mRNA was detected in tumours with homozygous deletion of the gene, whereas these cases expressed variable amounts of mRNA for both cdk4 and cyclin D1 (Figure 2 and Table I). Interestingly, although none of the 12 normal tissues examined expressed detectable levels of the *CDKN*2 transcript, 33 of the sarcomas (33%) showed high to very high mRNA levels ('++' or '+++'), of which 18 were patient biopsies (14 primary tumours and four metastatic lesions, i.e. 26% of the patient biopsies), whereas 13 originated from xenografts (48% of the xenografts) and two were *in vitro* cell lines (50%) (Table II).

All 12 tumours with *CDK*4 amplification demonstrated a high ('++' or '+++') transcript level, and 11 of these tumours showed a concomitant high expression of one of the two other genes (Figure 2 and Table I). Moreover, high ('++') *CDK*4 expression was found also in nine tumours without amplification of the gene (Table II). Altogether, elevated *CDK*4 transcript levels were present in biopsies from 11 patients (16%), in seven sarcoma xenografts (26%) and in three *in vitro* cell lines (75%). The expression levels in normal tissues varied. Thus, specimens obtained from kidney, lung, ovary and breast gland showed a high ('++') expression, whereas the other tissues demonstrated a low, but detectable ('+') *CDK*4 mRNA level.

Except for lung and skin, the mRNA level of *CCND*1 was generally low in the normal tissues. In contrast, high expression levels were found in 37 sarcomas (37%), of which 24 were tumour biopsies (35% of the patient biopsies), 12 xenografts (44%) and one of four cultured cell lines (Table II). Unlike the results of the other transcripts, the expression patterns of *CCND*1 differed in the two main groups of sarcomas. Thus, whereas 42% of the soft-tissue tumours

**Table I** Tumours with DNA[a] deletion or amplification affecting either of the *CDKN2*, *CDK4* or *CCND1* genes. Relationship to the mRNA levels[b]

| *Tumours[c] with DNA aberrations (total no. of tumours of each subtype)* | *CDKN2* | | *CDK4* | | *CCND1* | |
|---|---|---|---|---|---|---|
| | *DNA* | *mRNA* | *DNA* | *mRNA* | *DNA* | *mRNA* |
| Osteosarcoma ($n = 21$) | | | | | | |
| OS6x | N | +(+) | A | ++ | N | (−) |
| OS11p | D | − | N | +++ | N | + |
| OSAcl | N | +++ | A | +++ | A | +++ |
| Chondrosarcoma ($n = 2$) | | | | | | |
| CS2p | D | − | N | (+) | N | ++ |
| Fibrosarcoma ($n = 5$) | | | | | | |
| FS1p | N | ++ | A | +++ | N | + |
| FS2p | N | (+) | A | ++ | N | ++ |
| Haemangiopericytoma ($n = 1$) | | | | | | |
| HP1x | N | ++ | A | +++ | A | +++ |
| Leiomyosarcoma ($n = 13$) | | | | | | |
| LMS8p | D | − | N | (+) | N | +++ |
| Liposarcoma ($n = 21$) | | | | | | |
| LS2p | N | (+) | N | (+) | A | +++ |
| LS11p | N | ++ | A | +++ | N | +++ |
| LS21p | N | ++ | A | +++ | N | + |
| LS22p | N | (+) | A | ++ | N | ++ |
| LS28x | N | +++ | A | +++ | N | ++ |
| Malignant Schwannoma ($n = 8$) | | | | | | |
| MS2x | D | − | N | + | N | ++ |
| MS7p | D | − | N | ++ | N | + |
| MS8p | N | ++ | A | +++ | N | + |
| Rhabdomyosarcoma ($n = 5$) | | | | | | |
| RMS3p | D | − | N | + | N | − |
| RMS4cl | D | − | N | +++ | N | + |
| RMS13cl | N | +++ | A | +++ | N | ++ |
| Non-classified sarcoma ($n = 11$) | | | | | | |
| NCS2x | N | +++ | A | +++ | A | +++ |
| NCS7p | D | − | N | + | N | (+) |
| No. of tumours with DNA alterations | 8 | | 12 | | 4 | |

[a]N, normal; D, deletion; A, amplification. [b]Expression levels as described in Materials and methods. [c]cl, cell line; p, patient biopsy; x, xenograft. Altogether 109 tumours.

expressed high levels of cyclin D1 mRNA, only 22% of the osteogenic sarcomas did so.

### *Relationship between the mRNA levels of* CDKN2, CDK*4 and* CCND*1*

Of the 33 tumours found to express high levels of mRNA encoding the cdk4 inhibitor, 25 (76%) had low or undetectable level of the *CCND1* transcript. Conversely, of the 37 tumours with high *CCND1* expression, 29 (78%) did not express detectable amounts of the inhibitor mRNA. Taken together, these results suggest an inverse relationship between the mRNA levels of the kinase inhibitor and the kinase activator (two-sided Fischer exact test, $P = 0.08$).

In 10 of the 33 tumours the elevated inhibitor expression was accompanied by a high level of *CDK4* mRNA, including nine cases with amplification of the kinase gene. Only five tumours, all with amplification of *CDK4*, showed high expression of all three genes.

### Discussion

The *CDKN2* gene, encoding an inhibitor of cdk4 activity, was recently reported to be homozygously deleted in a high percentage of human cancer cell lines (Kamb *et al.*, 1994; Nobori *et al.*, 1994). Based on these results it was suggested that the inhibitor, p16, may be an important new tumour-suppressor protein (Kamb *et al.*, 1994). By binding to cdk4, p16 inhibits the formation of the cyclin D1–cdk4 complex, which is known to phosphorylate and thereby inactivate the retinoblastoma protein (Serrano *et al.*, 1993). The loss of p16 expression caused by gene deletion may result in increased cdk4-induced phosphorylation of pRb, thus releasing the $G_1$ cell cycle block.

To investigate whether *CDKN2* aberations might be involved in the tumorigenesis of human sarcoma, a panel of more than 100 tumours of various histological subtypes was analysed for homozygous deletions of the gene. It was found that only eight of the sarcomas showed such deletions. Notably, no deletion of *CDKN2* was seen in any of the 21 liposarcomas and 20 MFHs studied, and in only one of 21 osteosarcomas. The low deletion frequency stands in contrast to the 25–87% homozygous deletions found in various human tumour cell lines, including 60% of the human osteosarcoma cell lines studied (Kamb *et al.*, 1994; Nobori *et al.*, 1994). Interestingly, however, we found that two of eight malignant Schwannomas and two of five rhabdomyosarcomas had lost *CDKN2*, suggesting that homozygous deletions of *CDKN2* might be of different importance in the development of various sarcoma subtypes. The overall low incidence of deletions found here is in accordance with the results of Spruck *et al.* (1994) and Cairns *et al.* (1994), who observed homozygous deletions that included the *CDKN2* gene only in about 10–20% of bladder, brain, head and neck and lung carcinomas. Conceivably, the high deletion frequency observed in cell lines might be a result of *in vitro* cell cultivation. Our data do not permit any conclusions on this, but it was found that one of five sarcoma cell lines had lost the *CDKN2* gene, whereas only one of 27 (4%) sarcomas

**Figure 1** Representative Southern blot analysis demonstrating homozygous deletion of *CDKN*2 (**a** and **b**) and amplification of *CDK*4 and *CCND*1 (**a**). The DNA (7 μg in each lane), digested with *Hin*dIII, was subsequently hybridised with probes encoding the three different genes and, as a control, with an *APOB* probe. Samples with DNA aberrations, scored as described in Materials and methods, are indicated by a D (deletion) or A (amplification) below the corresponding panel. For the MS8 tumour, two additional bands emerged with the *CCND*1 cDNA probe owing to cross-hybridisation of cyclin D2 which is amplified in this tumour.

**Figure 2** Northern blot analysis demonstrating the mRNA levels of *CDKN*2, *CDK*4 and *CCND*1 in the same sarcomas as listed in Figure 1a and the OSA cell line. Five micrograms of total RNA in each lane was subsequently hybridised with probes encoding the three different genes, and as a control with an 18 S rRNA oligonucleotide probe.

grown as xenografts in nude mice harboured such lesions. When tumour material and xenografts established from the same patient could be examined, identical results on *CDKN*2 aberrations were obtained, indicating that the process of xenografting does not induce homozygous deletion of the gene, in accordance with the results of Caldas *et al.* (1994).

The possibility exists that the somewhat contradicting reports concerning the incidence of homozygous *CDKN*2 deletions might in part be related to methodological factors. Despite careful dissection of the tumour tissue before freezing, infiltration of normal cells in the tumour biopsy could result in a low observed deletion frequency. However, Southern blot analysis, as used here, should be less sensitive to a moderate contribution from normal tissue than the PCR analysis used by others (Cairns *et al.*, 1994; Kamb *et al.*, 1994; Nobori *et al.*, 1994; Spruck *et al.*, 1994). Furthermore, it should be noted that part of our sarcoma material has previously been analysed for loss of heterozygosity of the *TP*53 gene without any indications of normal cell DNA affecting the results (Andreassen *et al.*, 1993). It cannot be excluded that the *CDKN*2 gene may be inactivated by mechanisms other than loss of the entire gene, including point mutations and small deletions, as have been reported in cases of oesophageal (Mori *et al.*, 1994), non-

Table II Tumours with elevated mRNA expression

| Source of sample | No. analysed | No. of cases with high to very high mRNA levels[a] CDKN2 | CDK4 | CCND1 |
|---|---|---|---|---|
| Tumour tissue | 69 | 18 | 11 | 24 |
| Xenografts | 27 | 13 | 7 | 12 |
| Cell lines | 4 | 2 | 3 | 1 |
| Total | 100 | 33 (33%) | 21 (21%) | 37 (37%) |

[a]Scored as described in Materials and methods.

small-cell lung (Hayashi *et al.*, 1994) and pancreatic carcinomas (Caldas *et al.*, 1994). Thus, in order to reveal whether such abnormalities may be important in the tumorigenicity of sarcomas, the tumours will be analysed for mutations in exon 1 and 2.

The mechanism of action of the p16 inhibitor made it of interest to examine the tumour panel for aberrations also in the genes encoding cdk4 and cyclin D1. *CDK4* has previously been found amplified in sarcoma cell lines (Khatib *et al.*, 1993) and tumour biopsies (Forus *et al.*, 1995). Moreover, deregulation of the *CCND*1 gene has been observed in several cancer forms, caused either by tumour-specific gene translocation (Motokura and Arnold, 1993) or by gene amplification (Lammie *et al.*, 1991). *CDK*4 is located in the q13–14 region of human chromosome 12, and amplification of genes in this chromosomal segment has frequently been observed in human sarcomas (Forus *et al.*, 1993; Demetrick *et al.*, 1994). In the present work, *CDK*4 amplification was observed in 12 cases, and in three of these tumours *CCND*1 was also amplified. It might be speculated that there is a synergistic growth advantage when both genes are amplified. Amplification of *CDK*4 and/or of *CCND*1 found in the tumour biopsies was never accompanied by *CDKN*2 deletion. This is in accordance with the view that this growth-regulating pathway can be deregulated either by a loss of the inhibitor or by an increase in the amount of the rate-limiting cyclin D1/cdk4 complex. Most of our samples have been examined for amplification also of the two other D-type cyclins, cyclin D2 and cyclin D3, both able to form a complex with cdk4 in a similar way as cyclin D1 (Bates *et al.*, 1994). These genes were amplified in only one case each, both concomitant with amplification of *CDK*4 but not of *CCND*1 (results not shown).

The mRNA levels of *CDK*4, *CCND*1 and *CDKN*2 were examined in an attempt to detect possible consistent patterns of co-variation between the three transcripts, and to relate the results to the DNA status. Although caution must be taken when drawing conclusions on the activity of their associated gene products, it has been reported that the amount of cyclin D1–cdk4 complex varies in relation to the *CDK*4 mRNA levels (Pines, 1993) and that synthesis of cyclin D1, a protein with short half-life, is necessary and rate limiting for $G_1$ progression (Matsushime *et al.*, 1991; Baldin *et al.*, 1993; Hunter and Pines, 1994). Taken together, this indicates that the mRNA levels might be informative for the protein activities.

Interestingly, and surprisingly for a cell cycle inhibitor, 33% of our tumours showed large amounts of *CDKN*2 mRNA compared with undetectable levels in all normal tissue samples examined. Moreover, in 25 of the 33 cases, high inhibitor mRNA expression was accompanied by absent or only negligible levels of the *CCND*1 transcript. Conversely, most of the sarcomas with high cyclin D1 expression (37%) showed low expression of the inhibitor, suggesting an inverse relationship between these two transcripts. The tumour-promoting effects of p16, cdk4 or cyclin D1 aberrations are most likely due to their influence on the phosphorylation of the retinoblastoma protein, and thus on entry into S-phase. The cdk4 inhibitor is suggested to function as a negative regulator of cdk4 once pRb has been inactivated by phosphorylation (Serano *et al.*, 1993). In cells in which the pRb tumour-suppressor protein is constitutively inactivated, p16 expression is hypothesised to be elevated with a subsequent inhibition of the kinase (Serrano *et al.*, 1993). Furthermore, it has been shown that the *CCND*1 expression may be positively regulated at the transcriptional level by active, unphosphorylated pRb (Bates *et al.*, 1994; Hunter and Pines, 1994; Müller *et al.*, 1994). Therefore, it seems reasonable to assume that cells with low expression of *CCND*1 and elevated expression of *CDKN*2 may be pRb deficient, and studies to examine this hypothesis have been initiated.

Only 4 of the 37 sarcomas with elevated *CCND*1 mRNA levels had amplification of the gene. Similarly, in breast carcinomas elevated levels of cyclin D1 mRNA and protein have been found to occur without concomitant gene aberrations, suggesting that other molecular mechanisms may induce overexpression of the cyclin D1 gene (Buckley *et al.*, 1993; Bartkova *et al.*, 1994). Elevated cyclin D1 expression may overcome pRb-mediated growth inhibition, as has been demonstrated in oesophageal (Jiang *et al.*, 1993) and non-small-cell lung carcinomas (Schauer *et al.*, 1994). It has been speculated whether other mechanisms than cyclin D1–cdk4 mediated pRb phosphorylation may be involved, as no phosphorylated tumour-suppressor protein was detected when cyclin D1 and pRb were cotransfected into SaOS-2 cells (Hinds *et al.*, 1992; Dowdy *et al.*, 1993). Thus, it is possible that such mechanisms might help explain the high fraction of sarcomas expressing elevated levels of cyclin D1 mRNA.

In summary, although the overall frequency of homozygous deletions of *CDKN*2 was found to be low in sarcomas, the indication of a higher incidence of such deletions among the malignant Schwannomas and rhabdomyosarcomas suggests that inactivation of the gene may be of particular significance in the development of these sarcoma subtypes. The overexpression of *CDKN*2 observed in a considerable fraction of all the sarcomas studied might be related to pRb deficiency or to mechanisms stabilising the mRNA. Moreover, the high fraction of sarcomas with amplification and/or increased mRNA levels of *CDK*4 and *CCND*1 further supports the view that disturbance of this pathway may be of importance in the tumorigenesis of sarcomas.

### Abbreviations

*CDK*4, cyclin-dependent kinase 4; *CDKN*2, the cdk4 inhibitor, p16; *CCND*1, cyclin D1; *APOB*, apolipoprotein B; pRb, retinoblastoma protein; MFH, malignant fibrous histiocytoma, SDS, sodium dodecyl sulphate; SSC, standard saline citrate; PCR, polymerase chain reaction

### Acknowledgements

We thank Tove Øyjord for excellent technical assistance and we are indebted to Frances Jaques for her secretarial assistance. This work was supported by The Norwegian Cancer Society, The Anders Jahre Foundation and Rakel and Otto Bruuns legacy.

### References

ANDREASSEN A, ØYJORD T, HOVIG E, HOLM R, FLØRENES VA, NESLAND JM, MYKLEBOST O, HØIE J, BRULAND ØS, BØRRESEN AL AND FODSTAD Ø. (1993). p53 abnormalities in different subtypes of human sarcomas. *Cancer Res.*, **53**, 468–471.

BALDIN V, LUKAS J, MARCOTE M, PAGANO MJ AND DRAETTA G. (1993). Cyclin D1 is a nuclear protein required for cell cycle progression in G1. *Genes Dev.*, **7**, 812–821.

BARTKOVA J, LUKAS J, STRAUSS M AND BARTEK J. (1994). Cell cycle-related variation and tissue-restricted expression of human cyclin D1 protein. *J. Pathol.*, **172,** 237–245.

BATES S, PARRY D, BONETTA L, VOUSDEN K, DICKSON C AND PETERS G. (1994). Absence of cyclin D/cdk complexes in cells lacking functional retinoblastoma protein. *Oncogene*, **9,** 1633–1640.

BUCKLEY MF, SWEENEY KJ, HAMILTON JA, SINI RL, MANNING DL, NICHOLSON RI, DEFAZIO A, WATTS CKW, MUSGROVE EA AND SUTHERLAND RL. (1993). Expression and amplification of cyclin genes in human breast cancer. *Oncogene*, **8,** 2127–2133.

CAIRNS P, MAO L, MERLO A, LEE DJ, SCHWAB D, EBY Y, TOKINO K, RIET Pvd, BLAUGRUND JE AND SIDRANSKY D. (1994). Rates of p16 (*MTS*1) mutations in primary tumors with 9p loss. *Science*, **265,** 415–416.

CALDAS C, HAHN SA, DACOSTA LT, REDSTON MS, SCHUTTE M, SEYMOUR AB, WEINSTEIN CL, HRUBAN RH, YEO CJ AND KERN SE. (1994). Frequent somatic mutations and homozygous deletions of the p16 (*MTS*1) gene in pancreatic adenocarcinoma. *Nature Genet.* **8,** 27–32.

CHURCH GM AND GILBERT W. (1984). Genomic sequencing. *Proc. Natl Acad. Sci. USA*, **81,** 1991–1995.

DEMETRICK DJ, ZHANG H AND BEACH DH. (1994). Chromosomal mapping of human *CDK*2, *CDK*4, and *CDK*5 cell cycle kinase genes. *Cytogenet. Cell Genet.*, **66,** 72–74.

DOWDY SF, HINDS PW, LOUIE K, REED SI, ARNOLD A AND WEINBERG RA. (1993). Physical interaction of the retinoblastoma protein with human D cyclins. *Cell*, **73,** 499–511.

FEINBERG AP AND VOGELSTEIN B. (1983). A technique for radio labeling DNA restriction endonuclease fragments to high specific activity. *Anal. Biochem.*, **132,** 6–13.

FORUS A, FLØRENES V, MAELANDSMO GM, FODSTAD Ø AND MYKLEBOST O. (1995). 12q13-14 amplica in human sarcomas without *MDM*2 include *CDK*4, *SAS and GADD*153/*CHOP*. *Cytogenet. Cell Genet.* (in press).

FORUS A, FLØRENES VA, MAELANDSMO GM, MELTZER PS, FODSTAD Ø AND MYKLEBOST O. (1993). Mapping of amplification units in the q13–14 region of chromosome 12 in human sarcomas: some amplica do not include *MDM*2. *Cell Growth Different*, **4,** 1065–1070.

HAYASHI N, SUGIMOTO Y, TSUCHIYA E, OGAWA M AND NAKAMURA Y. (1994). Somatic mutations of the MTS (multiple tumor suppressor) 1/CDK41 (cyclin-dependent kinase-4 inhibitor) gene in human primary non-small cell lung carcinomas. *Biochem. Biophys. Res. Commun.*, **202,** 1426–1430.

HINDS PW, MITTNACHT S, DULIC V, ARNOLD A, REED SI AND WEINBERG RA. (1992). Regulation of retinoblastoma protein functions by ectopic expression of human cyclins. *Cell*, **70,** 993–1006.

HUNTER T AND PINES J. (1994). Cyclins and cancer. II. Cyclin D and CDK inhibitors come of age. *Cell*, **79,** 573–582.

JIANG W, KAHN SM, TOMITA N, ZHANG YJ, LU SH AND WEINSTEIN IB. (1992). Amplification and expression of human cyclin D gene in esophageal cancer. *Cancer Res.*, **52,** 2980–2983.

JIANG W, ZHANG YJ, KAHN SM, HOLLSTEIN MC, SANTELLA RM, LU SH, HARRIS CC, MONTESANO R AND WEINSTEIN IB. (1993). Altered expression of the cyclin D1 and retinoblastoma genes in human esophageal cancer. *Proc. Natl Acad. Sci. USA*, **90,** 9026–9030.

KAMB A, GRUIS NA, WEAVER-FELDHAUS J, LIU Q, HARSHMAN K, TAVTIGIAN SV, STOCKERT E, DAY III RS, JOHNSON BE AND SKOLNICK MH. (1994). A cell cycle regulator potentially involved in genesis of many tumor types. *Science*, **264,** 436–440.

KHATIB ZA, MATSUSHIME H, VALENTINE M, SHAPIRO DN, SHERR CJ AND LOOK AT. (1993). Coamplification of the *CDK*4 gene with *MDM*2 and *GLI* in human sarcomas. *Cancer Res.*, **53,** 5535–5541.

LAMMIE GA, FANTL V, SMITH R, SCHUURING E, BROOKES S, MICHALIDES R, DICKSON C, ARNOLD A AND PETERS G. (1991). *D11S*287, a putative oncogene on chromosome 11q13, is amplified and expressed in squamous cell and mammary carcinomas and linked to *BCL*-1. *Oncogene*, **6,** 439–444.

MANIATIS T, FRITSCH EF AND SAMBROOK J. (1982). *Molecular Cloning: A Laboratory Manual.* Cold Spring Harbor Laboratory Press: Cold Spring Harbor, NY.

MATSUSHIME H, ROUSSEL RA, ASHMUN RA AND SHERR CJ. (1991). Colony-stimulating factor 1 regulates novel cyclins during the G1 phase of the cell cycle. *Cell*, **65,** 701–713.

MORI T, MIURA K, AOKI T, NISHIHIRA T, MORI S AND NAKAMURA Y. (1994). Frequent somatic mutation of the *MTS*1/*CDK*41 (multiple tumor suppressor/cyclin-dependent kinase 4 inhibitor) gene in esophageal squamous cell carcinoma. *Cancer Res.*, **54,** 3396–3397.

MOTOKURA T AND ARNOLD A. (1993). Cyclin D and oncogenesis (Review). *Curr. Opin. Genet. Dev.*, **3,** 5–10.

MÜLLER H, LUKAS J, SCHNEIDER A, WARTHOE P, BARTEK J, EILERS M AND STRAUSS M. (1994). Cyclin D1 expression is regulated by the retinoblastoma protein. *Proc. Natl Acad. Sci. USA*, **91,** 2945–2949.

NOBORI T, MIURA K, WU DJ, LOIS A, TAKABAYASHI K AND CARSON DA. (1994). Deletions of the cyclin-dependent kinase-4 inhibitor gene in multiple human cancers. *Nature*, **368,** 753–756.

PINES J. (1993). Cyclins and cyclin-dependent kinases: take your partners. *Trends Biochem. Sci.*, **18,** 195–197.

SCHAUER IE, SIRIWARDANA S, LANGAN TA AND SCLAFANI RA. (1994). Cyclin D1 overexpression vs. retinoblastoma inactivation: Implications for growth control evasion in non-small cell and small cell lung cancer. *Proc. Natl Acad. Sci. USA*, **91,** 7827–7831.

SERRANO M, HANNON GJ AND BEACH D. (1993). A new regulatory motif in cell-cycle control causing specific inhibition of cyclin D/CDK4. *Nature*, **366,** 704–707.

SPRUCK III. CH, GONZALEZ-ZULUETA M, SHIBATA A, SIMONEAU AR, LIN M-F, GONZALES F, TSAI YC AND JONES PA. (1994). p16 gene in uncultured tumours. *Nature*, **370,** 183–184.